# Cognitive Effect Following a Blended (Face to Face and Videoconference-Delivered) Format Mindfulness Training

**DOI:** 10.3389/fpsyg.2021.701459

**Published:** 2021-07-30

**Authors:** Grace Y. Wang, Tamasin Taylor, Alexander Sumich, Chris Krägeloh, Carol Qinglian Lee, Richard J. Siegert

**Affiliations:** ^1^Department of Psychology and Neuroscience, School of Clinical Sciences, Auckland University of Technology, Auckland, New Zealand; ^2^Faculty of Medical and Health Sciences, University of Auckland, Auckland, New Zealand; ^3^Division of Psychology, School of Social Sciences, Nottingham Trent University, Nottingham, United Kingdom

**Keywords:** mindfulness, cognitive function, e-therapy, webinar, attention

## Abstract

While evidence supports the feasibility of online mindfulness training (MT), the effect of this approach on cognition remains unclear. The present study investigated changes in cognition following a newly developed 6-week videoconference-delivered MT program on cognitive function in two groups. The first group (*n* = 17) had two baseline assessments prior to MT [3 weeks after group two (*n* = 15)] to allow for evaluation of practice and learning effects. Four participants from each group were excluded from the final analysis due to missing data. Following MT, there was an improvement in switching of attention, working memory, executive function, and social cognition, but some of these effects were not easily accounted for by learning or practice effects. No significant changes were found on tasks measuring sustained attention, cognitive flexibility and inhibition, information processing, and sensory-motor function. Our findings suggest that domain-specific cognition might be enhanced by a brief videoconference-delivered MT, and larger, controlled studies to delineate the effects of online MT on subdomains of cognition are needed.

## Introduction

Extensive evidence supports that mindfulness training (MT) improves both mental health and cognitive performance (Gu et al., 2015; Morrison and Jha, 2015). The mechanisms underlying the therapeutic effects of MT on psychological outcomes are primarily correlated with development of insight and non-reactive acceptance of one’s moment-to-moment experience (Kabat-Zinn, 2013). Over the last decades, communication technology has increasingly been applied to deliver mindfulness interventions or support mindfulness practice, ranging from phone-delivered sessions and mobile applications, to online websites or a combination of a virtual online classroom and website (Wahbeh et al., 2014; Gu et al., 2018). Given improved digital accessibility, online MT is considered a more convenient and cost-effective strategy, compared to traditional face-to-face interventions (Jayawardene et al., 2017). Furthermore, the recent COVID-19 pandemic has caused a revolutionary uptake in the use of telehealth in mental health care delivery. This need for flexible healthcare delivery formats is projected to remain after the pandemic has ended (Pierce et al., 2021). A meta-analysis showed the comparable positive effects of online MT on stress, depression, anxiety, and wellbeing results to traditional interventions (Spijkerman et al., 2016).

While evidence supports the benefit and feasibility of online MT, the effect of online MT on cognition appears unclear. Some reported cognitive improvements associated with online MT, such as attention (Spadaro and Hunker, 2016; Bennike et al., 2017; Polsinelli et al., 2020) and socioemotional regulation (Davis and Zautra, 2013), while others found no online MT-related improvements in executive function and critical thinking (Noone and Hogan, 2018). The MT included in these studies were predominantly delivered through either websites or a smartphone application, and inconsistent findings might be related to unskilled practice due to a lack of guided practice of mindfulness meditation (Noone and Hogan, 2018). It is unrealistic to expect individuals to learn how to practice mindfulness just by using a smartphone application and without teachers involvement (Crane et al., 2012). It has been suggested that the use of technologies can support and improve the connectedness between individuals, groups, and organizations, and promote positive functioning, but the design needs to be guided by the scientific and applied approach, i.e., positive psychology, for users’ experiences (Riva et al., 2012). For example, improvement in positive emotional states is significantly correlated to the users’ sense of presence during media exposure (i.e., video, audio, and virtual reality) (Villani et al., 2007; Navarro-Haro et al., 2017).

As an alternative to moving to an entirely technology-delivered format, various blended approaches have been proposed (Montero-Marin et al., 2018). These combine traditional face-to-face and online delivery of mindfulness interventions and could be perceived as a compromise that does not pose such a stark contrast to conventional face-to-face delivery and enhances acceptability for people who may still feel uncomfortable engaging in fully online group programs. For others who are happy with online formats, some may additionally appreciate having met the facilitator in person before continuing with the program online. For example, the face-to-face interaction could help to increase participants’ engagement in the training, and allow participants to talk about their concerns with the trainer and get the answers to their questions in real-time, which might lead to better outcome of MT.

As part of a larger study investigating the relationships between mindfulness, psychological well-being with brain and immune function (Wang et al., 2017; Doborjeh et al., 2019, 2020), we have recently shown that a 6-week MT delivered via a blended online setting – face-to face and video-conference – improves mindfulness, mood (depression, anxiety), and emotional bias (negative bias, dysfunctional attitude, poor self-compassion, and poor compassion for others), supporting this method as viable alternative format to standard mindfulness programs (Krägeloh et al., 2018). The first session of our MT program was delivered by the facilitator in person to ensure good rapport between facilitator and participants. Following this, the facilitator delivered the group MT remotely from the second session onward. In terms of content and structure, the intervention can be considered equivalent to standard mindfulness-based interventions where a range of mindfulness skills are taught (Krägeloh et al., 2019).

The present study investigated the short-term effect of this newly developed MT on cognitive function in healthy participants. The Liverpool Mindfulness Model (Malinowski, 2013) suggests that a simple form of MT could increase the efficiency of attentional functions and the behavioral improvements as well as changes in neural activity and underlying neural architecture subsequently lead to positive effects in various situations, and for various conditions. Thus, it was hypothesized that the participants would exhibit improved cognitive performance following MT, particularly on tasks related to attention control. We also expected to find improved memory and executive function which are often reported to be enhanced following the traditional face-to-face MT, i.e., (Jha et al., 2007; Morrison and Jha, 2015).

## Materials and Methods

### Participants

Participants were recruited via the university through various advertisements on posters, at lectures, websites, and student services. People with a self-reported history of traumatic brain injury, alcohol or substance abuse, epilepsy, psychotic disorders, and those on medications for acute mental illnesses (e.g., major depressive disorder, anxiety disorder, and schizophrenic disorder) were excluded. Moreover, to avoid potential conflicts of interest or dual roles, students of research team members were also excluded. During the recruitment, a total of 204 individuals expressed their interest in participating in the research, of which three did not meet the inclusion criteria of absence of psychological condition requiring ongoing medication, absence of epilepsy or brain injury, and no alcohol of substance abuse. Of those remaining, 42 initially confirmed their participation in the study, but a further ten were subsequently not able to continue. Thus, the final sample size in the present study was 32 (*n* = 21 women) participants, 18–58 years (mean age = 30.06, SD = 11.27). The participants were then randomly allocated to either the group one (*n* = 15) or the group two (*n* = 17). The ethnic makeup was diverse, including New Zealand European (*n* = 14; 44%), Asian (*n* = 6; 19%), Indian (*n* = 3; 9%), Mâori (*n* = 1; 3%), Pacific Islander (*n* = 1; 3%), and others or not specified (*n* = 7; 22%). There were no significant differences between groups in the demographic profiles, including age, gender, and ethnicity (*p* > 0.05).

Based on previous research that has demonstrated significant changes in attention following an online MT with an effect size ranging from 0.30 to 0.70 (Spadaro and Hunker, 2016; Polsinelli et al., 2020), it was calculated using G^∗^Power 3.1 (Faul et al., 2009) that a minimum sample size of 12 participants per group would have 80% power to detect an expected effect size of *d* = 0.40 with a two-tailed α of 0.05 for a 1 (group) by 3 (time) mixed repeated-measures ANOVA within factors (primary outcome: attention).

### Mindfulness Training

The MT was led by an experienced facilitator, who has been practicing mindfulness for 20 years and is considered one of the leading mindfulness practitioners in New Zealand.^1^ The facilitator was blinded to the study hypothesis and was not involved in study design. The duration of the training was 6 weeks with a weekly session that ran for between 90 and 110 min. Adverse effects were monitored by a clinical psychologist who also participated in the exercises but identified herself as staff who was able to help if any of the students were experiencing any psychological distress during and after sessions.

The facilitator conducted the first session face-to-face in a quiet university room. Subsequent sessions were conducted in the same room with participants present in a group and the facilitator present via a live Internet-based video conference webinar. The first session included an introduction exercise, explanations of the basic principles of mindfulness and the purpose of the course, and a guided meditation for 10 min. Session 2 and beyond was presented via a webinar using GoToMeeting (commercially available videoconferencing software). Session 2 comprised of approximately 10–15 min of physical exercise comparable to Taijichuan, breathing meditation exercises, and a PowerPoint presentation on the brain and mindfulness. The session concluded with a brief guided meditation exercise and a mindful eating exercise. Session 3 composed of the same guided breathing meditation and light physical exercise, together with the presentation on the types of awareness, negative bias, and advantages of walking meditation. Session 4 included physical breathing exercises, breathing meditation, and a brief discussion on the fundamentals of mindfulness and emotion. Parallel to session 3, this session similarly concluded with a meditation exercise, particularly focusing on observing sound, body, and emotion. Session 5 comprised of physical movement exercise, concentration meditation, and a discussion on accepting and regulating emotions. Session 6 involved meditation practice, physical movement and breathing exercises, and a discussion on the four foundations of mindfulness and their purpose. All sessions finished with an opportunity for participants to ask the facilitator questions. The exercises were either covered in the course or demonstrated in an online video with a hyperlink sent to participants following each session. Participants were encouraged to practice for at least 15 min per day. Exercises to be practiced were either those covered in class or those shown by following a link to audio or video file sent to the participants after each session. A reminder email as well as a link to an online questionnaire inquiring about their home practice during that week were sent to participants a day before the start of the next session.

### Assessment of Cognitive Function

A computerized neuropsychological test battery – IntegNeuro (Brain Resource Company, Australia), was used to assess cognitive function across different time points of MT. This test battery has sound convergent and divergent validity as shown by strong associations with corresponding standard paper-and-pencil batteries (Paul et al., 2005). The reliability coefficient across all tests and measures ranges from medium to high (Williams et al., 2005). In the current study, 14 tasks were used to evaluate: sensory-motor function, information processing, attention, memory, language, executive function, cognitive inhibition, and social cognition (Table 1). Further details of each of the tests in this battery can be found in Wang et al. (2014). The tasks we used were expected to be sensitive to the effects of a brief MT which have been shown in previous studies (Moore and Malinowski, 2009; Zeidan et al., 2010), and also allow some latitude for exploration of MT-related cognitive effects that are still less-investigated and remain largely unclear, e.g., sensor-motor function, language, and social cognition.

**TABLE 1:** Description of neuropsychological measures.

Cognitive domains	IntegNeuro test	Equivalent to traditional tasks	Time to complete
Attention	Time estimation		2 min
	Continuous performance	Test of variables of attention (399) and conners continuous performance task (400)	6 min
	Switching of attention	Trail making test Part A & B (Reitan, 1958)	4 min
Cognitive flexibility	Verbal interference	Stroop task (Stroop, 1935)	3 min
Cognitive inhibition	Go-NoGo		6 min
Executive function	Maze	Austine maze (Walsh, 1985)	8 min
Information processing	Choice reaction time	Corsi blocks test (Milner, 1971)	3 min
Language	Spot the real word	National adult reading test (Nelson and Willison, 1991) and spot the real word intelligence test (Baddeley et al., 1993)	2 min
	Word generation	Controlled oral word test (Benton et al., 1994)	6 min
Memory	Span of visual memory	Corsi blocks test (Milner, 1971).	5 min
	Digit span	Weschler adult intelligence scale (WAIS)-III digit span test (Weschler, 1997)	5 min
	Memory recall and recognition	Rey auditory verbal learning test (Rey, 1941) and the California learning and verbal memory (Delis et al., 1987)	7 min
Sensori-motor	Motor tapping	Finger tapping test (Wertham, 1929)	2 min
Social cognition	Emotion recognition	Penn emotion recognition test (Kohler et al., 2003)	10 min

### Procedure

Ethics approval was granted by the authors’ institutional Ethics Committee (Reference Number16/147). Written consent was obtained from each participant prior to data collection. The first group had an initial baseline followed by a 3-week lead-in, a second baseline assessment (Pre-MT), and MT follow-up assessment (post-MT, within 1 week following final MT session). The second group started the 6-week mindfulness program 3 weeks prior to the first group. The second group had a single baseline assessment, followed immediately by commencement of MT, an initial follow-up (post-MT, within 1 week following final MT session) and a second (3 weeks follow-up) post-intervention assessment. This design allowed us to explore history and learning effects as well as allowing a longer-term follow-up. It also allowed the wait-listed participants an opportunity to obtain the MT at a later date. However, there were differences in the time span between the assessments of cognitive tasks in two groups due to the nature of tertiary education year, such as mid-term breaks and exam periods. The actual day and time were arranged according to the participants’ availability. The MT sessions for the two groups followed the same outline. Participants were given a $20 gift voucher each time they were tested. The same procedures and order of cognitive subtests were followed for each testing session.

### Data Analysis

The population was presumed to be non-normally distributed due to small sample size (Shapiro–Wilk test, s-w = 0.43–0.76, *p* < 0.01), thus, MT-related cognitive changes across time in each group were examined, respectively, using a Friedman test, respectively. Given that multiple comparisons were involved, a Benjamini–Hochberg procedure was applied for adjusting the false discovery rate (*p*-value ≤ 0.02). Benjamini–Hochberg procedures is considered a powerful method of controlling the false discovery rate which involves sorting the *p*-values in order from small to large and rejecting the proportion of significant results that are considered false positives (Benjamini and Hochberg, 1995; Glen, 2015; Chen et al., 2017). The chosen false discovery rate was 0.20, with 80% chance to detect significant differences after correction. When there was a significant time effect in the Friedman test, changes in cognitive performance at each time point were assessed using a Mann–Whitney Wilcoxon test.

To test the possible group^∗^time interaction, we further conducted linear mix-effect models with the combination of the two groups for cognitive domains that showed significant changes following the MT, based on the results of Mann–Whitney Wilcoxon test. Due to differences in time span of testing sessions between groups, we only included cognitive test scores assessed at baseline and post MT. In each model, time was included as a fixed effect and Group as a covariate in order to detect the MT effect on cognition, controlling for possible group allocation effect. We also conducted additional models to test whether group allocation (i.e., the first group had a 3-week lead-in prior to receive MT) interacted with MT to influence cognitive functioning. The group^∗^time interactions were non-significant (*p* > 0.05). As noted above, the first group also received the intervention, although delayed. The lack of interaction therefore does not indicate a lack of an intervention effect but lack of order effect that may be related to group allocation. All analyses were two-tailed, and the probability level of *p* < 0.05 indicated statistical significance. Analyses were performed using IBM SPSS Statistics (version 22).

## Results

A summary of participants’ cognitive performance for the two groups is reported in the Supplementary Material. No significant group differences in any of the cognitive tests were found at baseline.

### Group One (Baseline vs. Pre-MT vs. Post-MT)

Of the 17 participants, four did not complete post-MT testing. Of the remaining participants (*n* = 13), 12 completed at least four MT sessions and one person completed three sessions.

Friedamn’s tests showed that there were significant change in task performance over time in tasks measuring immediate memory recall (*χ*^2^ = 8.21, *p* = 0.02), executive function (Maze error: *χ*^2^ = 16.67, *p* < 0.001), and language (Word generation: *χ*^2^ = 17.17, *p* < 0.001). *Post hoc* tests using a Wilcoxon signed-rank test suggested some practice/learning effects were present prior to the MT as better performance was seen at the pre-MT in immediate recall (*z* = −2.71, *p* = 0.007), executive function (Maze error: *z* = −2.32, *p* = 0.02) and language (*z* = −3.07, *p* = 0.002) compared to baseline (Table 2). Nevertheless, improvement in executive function and language remained following the MT suggested by the participants’ superior performance post-MT relative to either pre-MT or at baseline.

**TABLE 2:** A summary of *post hoc* test results for group one.

Cognitive domain	Test	Pre-MT vs. baseline		Post-MT vs. pre-MT		Post-MT vs. baseline		Mean rank
		*z*	*p*	*z*	*p*	*z*	*p*	
Memory	Immediate memory recall	**−2.71**	**0.007**	−1.07	0.28	**−2.56**	**0.01**	Baseline < Pre-MT
								Pre-MT = Post-MT
								Baseline < Post-MT
Executive function	Maze: error	**−2.32**	**0.02**	**−1.96**	**0.05**	**−3.06**	**0.002**	Baseline < Pre-MT
								Pre-MT < Post-MT
								Baseline < Post-MT
Language	Word generation	**−3.07**	**0.002**	**−2.44**	**0.02**	**−2.94**	**0.003**	Baseline < Pre-MT
								Pre-MT < Post-MT
								Baseline < Post-MT

*< refers to improved performance; = refers to no significant difference in performance; Bold indicates significant results.*

### Group Two (Baseline vs. Post-MT vs. Follow-Up)

Among 15 participants, one person missed baseline cognitive testing, and three people missed post-MT testing. These people were excluded from final analysis. Of the remaining 11 participants, all attended at least four session of MT apart from one who attended only three.

Friedamn’s tests showed that there were significant changes in cognitive performance over time on several domains, including attention (Switch of attention: *χ*^2^ = 16.7, *p* < 0.001), memory (Immediate recall: *χ*^2^ = 11.82, *p* = 0.003), executive function (Maze completion time: *χ*^2^ = 7.94, *p* = 0.02; Maze error: *χ*^2^ = 7.60, *p* = 0.02) and Social cognition (Fear reaction time: *χ*^2^ = 10.5, *p* = 0.005). Nevertheless, there were no other significant effects associated with MT in the experimental group for the remaining cognitive tasks.

*Post hoc* tests showed that the performance of immediate recall was significantly improved post-MT (*z* = −2.55, *p* = 0.01) and at 3 week follow-up (*z* = −2.36, *p* = 0.02) relative to the baseline, suggesting the potential positive effect of MT in memory (Table 3). In terms of attention, less time was taken to complete the Switch of attention task following MT compared to the baseline, but the change was not significant (*z* = −1.48, *p* = 0.14); however, the improved performance reached significant at the 3-week Follow-up (*z* = −2.67, *p* = 0.008). In the maze task, fewer errors were made at post-MT (*z* = −2.10, *p* = 0.04) and at 3-weeks follow-up (*z* = −2.38, *p* = 0.02), relative to baseline, suggesting consistent improvement in executive function. Furthermore, significantly reduced Fear reaction time was found at 3-week follow-up compared to both baseline (*z* = −2.31, *p* = 0.02) and the post-MT (*z* = −2.50, *p* = 0.01), although no significant difference was seen between baseline and the post-MT (*z* = −1.24, *p* = 0.21).

**TABLE 3:** A summary of *post hoc* test results for group two.

Cognitive domain	Test	Post-MT vs. baseline		Follow-up vs. post-MT		Follow-up vs. baseline		Mean rank
		*z*	*p*	*z*	*p*	*z*	*p*	
Memory	Immediate memory recall	**−2.55**	**0.01**	−1.42	0.16	**−2.36**	**0.02**	Baseline < Post-MT
								Baseline < Follow-up
								Follow-up = Post-MT
Attention	Switch of attention	−1.48	0.14	**−2.67**	**0.008**	**−2.67**	**0.008**	Baseline = Post-MT
								Baseline < Follow-up
								Post-MT < Follow-up
Executive function	Maze: errors	**−2.10**	**0.04**	−1.42	0.15	**−2.38**	**0.02**	Baseline < Post-MT
								Baseline < Follow-up
								Post-MT = Follow-up
Social cognition	Fear reaction time	−1.24	0.21	**−2.50**	**0.01**	**−2.31**	**0.02**	Baseline = Post-MT
								Baseline < Follow-up
								Post-MT < Follow-up

*< refers to improved performance; = refers to no significant difference in performance; Bold indicates significant results.*

### Group Differences: Fixed-Effect Contributions

Controlling for the group effect, there were significant effect of time on switching of attention (Estimate = 5040.99, Std. Error = 2354.6, *t* = 2.14, and *p* = 0.04), memory (Estimate = 4.54, Std. Error = 0.90, *t* = 5.01, and *p* < 0.001), and executive function (Estimate = 54204.5, Std. Error = 2542, *t* = 2.13, and *P* = 0.04), suggesting improved cognitive performance regardless group allocations, e.g., receiving MT 3 weeks late in the first group.

## Discussion

Previous studies have found significant improved attention, executive function, and memory recall associated with MT (Chambers et al., 2008; Jha et al., 2010; Zeidan et al., 2010; Mrazek et al., 2013), suggesting the cognitive enhancement associated with MT. However, the effect of online MT on cognitive function appears unclear. The aim of this study was to investigate the effect of a newly developed blended MT program on a wide range of cognitive domains. Our results showed that there were significant improvements in attention switching, executive function, memory recall, language, and social cognition following MT. Some of these improvements, i.e., Maze and executive function, even remained 3 weeks after the MT. These findings are in agreement with previous studies which have also found significant improved attention, executive function, memory recall, language, and social cognition associated with MT (Chambers et al., 2008; Jha et al., 2010; Zeidan et al., 2010; Mrazek et al., 2013), suggesting the cognitive enhancement associated with MT. Importantly, the observed changes in attention, memory, and executive function were unlikely to be due to type-1 error given alpha adjustment and observed significance in these domains from the linear mixed-effect modes. However, it is worth nothing that the practice/learning effects might also be involved in the observed positive results, in particular for memory, language, and executive function. Additionally, there were variations in MT-related cognitive effects for groups. For example, the second group did not exhibit significant changes in the attention and social cognition tasks compared to the first group following MT, although their performance appeared to improve over the study period (Tables 2, 3). This might suggest that implementing time-variable training affects treatment outcome on cognition. Health research suggests that waiting time effects may exceed treatment effects (Minder et al., 2018) and a moderate decline in patient outcomes 12 months after treatment acceptance for additional days of waiting has been reported (Reichert and Jacobs, 2018). Furthermore, there are potential effects of the time in which the tests were administered to the participants on observation of task performance change. For example, in the present study, comparing post-MT with baseline cognitive measures was completely different from comparing post-MT with follow-up.

Contrary to previous aforementioned research (Jha et al., 2007; Heeren et al., 2009; Zanesco et al., 2013), we did not find significant changes in cognitive flexibility/inhibition, information processing, and sensory-motor function following MT, suggesting a lack of short-term effects of MT on these particular domains in the current samples. As a core component of mindfulness is related to inhibitory control, the absence of a positive effect on cognitive flexibility/inhibition was unexpected. Nevertheless, no significant changes in cognitive flexibility and information processing following MT have also been reported by others, e.g., (Wahbeh et al., 2016; Oken et al., 2017). It was suggested that the absence of MT-related effects on some of the cognitive domains may be related to the small sample size and a short training period (Wahbeh et al., 2016), or the ceiling effect related to cognitive performance at baseline (Oken et al., 2017).

In general, cognition requires the interplay of anatomically separated and interconnected local networks (Dajani and Uddin, 2015). Thus, observed improvement in specific domain may reflect a better interaction between cognitive networks. For example, executive function is a collection of cognitive processes which includes, but are not limited to, working memory, attention, cognitive flexibility, and impulse control (Logue and Gould, 2014). Evidence suggests that MT increases the capacity for effective emotion regulation such as openness and sensitivity to subtle changes in affective states, and these subtle changes in affective states fosters better executive control (Teper et al., 2013).

The Liverpool Model proposes a central role of attentional control in the development of mindfulness skills, and suggests that simple meditation practice, such as mindfully focusing on the somatosensory experiences of breathing, exerts a positive influence on attentional functions by improving resource allocation processes (Malinowski, 2013). Furthermore, improved positive subjective affect associated with MT could also influence how individuals process information and allocate attention. Evidence suggests that positive emotions encourage adaptive responses to environment and feeling good facilitates creativity, broadening of attentional scopes (Shiota et al., 2017). Thus, observed cognitive improvements are more likely to be found in the domains that are closely related to attentional control. Consistent with this notion, improvements in switching of attention, memory, and executive function following MT were found in the present study. In contrast, cognition underlying information processing, social cognition, and sensory-motor function may be less engaged or harder to be modified during a short MT. Further neuroimaging research with consideration of the effect of positive emotions, as well as the as sense of presence is required to clarify this.

Interestingly, we noticed that there was an increased reaction time on the Go-NoGo task following MT (Tables 2, 3). Although the changes did not reach statistical significance, it suggests a trend of slower reaction to the “go” signal. A study by van den Hurk et al. (2010) also found that mindfulness meditators exhibited slower motor response than their matched controls. It has been argued that such “slowness” is an intrinsic trait in a meditator who has high decision boundary, favoring accuracy and taking greater time to accumulate evidence before deciding (van Vugt and Jha, 2011). Given that our participants were not considered to be experienced mediators, it might be possible that our participants simply took more time to respond for improved accuracy.

It is also important to highlight that interventions appeared to be effective in improving psychological well-being might not always lead to observable cognitive enhancement. Our previous study have shown enhanced psychology well-being following this blended approach of combining face-to-face and online delivery of MT (Krägeloh et al., 2018), however, significant improvements were only found in some cognitive domains with this treatment.

There are limitations in our study and the present findings need to be interpreted with caution. Our study was limited by its sample size, a relatively short training timeframe (6 weeks), and participants’ adherence. Although we have made an effort to measure participants’ home practice, due to the low response rate and limited range of variability in scores, frequency and length of home practice could not be used in the present study as a co-variate. Furthermore, differences in the time span between the assessments of cognitive tasks in our groups might compromise the reliability of group comparison findings. The blended model for delivering the MT is also relatively new and a lack of real-time interaction during webinar sessions may compromise the actual effect of MT on cognition. Nevertheless, our findings demonstrate the potential of using the videoconference-delivered group format in MT.

## Conclusion

In conclusion, our findings indicate that MT could lead to improved cognitive functions over a brief 6-week course, and this effect is more apparent in some cognitive domains than others; specifically, attention switching, working memory, executive function, language, and social cognition. To date, the development of online interventions is still in its infancy and the question concerning whether the delivery methods diminish impact remains. Despite the study limitations, our findings highlight the subtle link between online MT and cognition which contributes to further understanding and application of MT in various formats. Given the potential value and increased acceptability of online MT for clinical practice, further research related to moderators of treatment effectiveness is warranted.

## Data Availability Statement

Raw data of this study are available from the corresponding author on request.

## Ethics Statement

The studies involving human participants were reviewed and approved by AUT Ethics Committee. The patients/participants provided their written informed consent to participate in this study.

## Author Contributions

RS led the funding application for this project. AS, CK, GW, and RS planned and designed the study. TT coordinated the recruitment and data collection. GW and CL analyzed the data. GW drafted the manuscript. All authors contributed to the manuscript and approved the final version.

## Conflict of Interest

The authors declare that the research was conducted in the absence of any commercial or financial relationships that could be construed as a potential conflict of interest.

## Publisher’s Note

All claims expressed in this article are solely those of the authors and do not necessarily represent those of their affiliated organizations, or those of the publisher, the editors and the reviewers. Any product that may be evaluated in this article, or claim that may be made by its manufacturer, is not guaranteed or endorsed by the publisher.

## Footnotes

1
https://mindfulnesseducation.nz/about-us/
