# Supplementary material for: Cognitive Effect Following a Blended (Face to Face and Videoconference-Delivered) Format Mindfulness Training
Source: Front Psychol. 2021 Jul 30;12:701459. doi: 10.3389/fpsyg.2021.701459 (PMC8360837; doi:10.3389/fpsyg.2021.701459)
Supplement: Supplementary file 1 [file Table_1.DOCX]

**Supplemental material:**

**A summary of cognitive test performance for groups.**

| Cognitive domain | Test | Measures | Group 2 | | | | | Group 1 | | | | |
| --- | --- | --- | --- | --- | --- | --- | --- | --- | --- | --- | --- | --- |
|  |  |  | Pre-MT | Post-MT | Follow-up | *χ^2^* value | *p* value | Baseline | Pre-MT | Post-MT | *χ^2^* value | *p* value |
|  |  |  | Mean (SD) | Mean (SD) | Mean (SD) |  |  | Mean (SD) | Mean (SD) | Mean (SD) |  |  |
| Attention | Continuous Performance | Reaction time (s) | 0.8 (0.2) | 0.7.4 (0.2) | 0.8(0.2) | 2.60 | 0.27 | 0.7(0.2) | 0.7 (0.2) | 0.8(0.2) | 1.50 | 0.47 |
|  |  | False alarm errors | 7.9 (16.1) | 2.3 (4.9) | 0.2(0.6) | 2.96 | 0.23 | 14.8 (27.9) | 8.4 (23.1) | 0.8(1.3) | 0.07 | 0.96 |
|  |  | False miss errors | 1.4 (1.6) | 1.4 (1.6) | 0.7(0.8) | 2.07 | 0.36 | 0.8 (1.0) | 1.4 (2.6) | 0.9(1.7) | 0.26 | 0.88 |
|  | Switching of Attention | Completion time (digits) (s) | 20.1 (6.6) | 17.98 (4.3) | 19.1(5.7) | 4.15 | 0.13 | 23.3 (9.5) | 18.9 (3.4) | 18.2(7.2) | 5.16 | 0.08 |
|  |  | Completion time (digits & letters) (s) | 48.0 (15.5) | 44.2 (11.7) | 38.4(12.8) | **16.7** | **<0.001** | 46.8 (17.9) | 42.7 (15.5) | 41.5(9.5) | 0.67 | 0.72 |
|  | Time Estimation | Accuracy | -0.06(0.1) | -0.04 (0.1) | -0.02(0.1) | 2.40 | 0.30 | -0.99(0.1) | -0.82 (0.1) | -0.79(0.1) | 2.17 | 0.33 |
| Cognitive flexibility | Verbal Interference | Accuracy (congruent) (%) | 15.9 (3.6) | 18.8 (3.6) | 19.3(3.7) | 6.06 | 0.05 | 16.9 (3.0) | 17.3 (4.6) | 17.4(3.0) | 0.21 | 0.90 |
|  |  | Reaction time (congruent (s) | 1.2(0.5) | 1.1(0.3) | 1.0(0.1) | 6.62 | 0.04 | 1.1(0.2) | 1.1(0.3) | 1.0(0.2) | 2.60 | 0.27 |
|  |  | Accuracy (incongruent) (%) | 14.1 (2.3) | 15.7 (3.9) | 15.8(3.9) | 4.41 | 0.11 | 13.3 (4.9) | 14.8 (2.5) | 13.9(3.8) | 2.05 | 0.36 |
|  |  | Reaction time (congruent (s) | 1.4(0.2) | 1.3(0.3) | 1.2(0.3) | 3.13 | 0.21 | 1.5(0.6) | 1.3(0.3) | 1.4(0.5) | 3.17 | 0.21 |
| Cognitive inhibition | Go-NoGo | False alarm errors (NoGo) | 2.2 (0.83) | 1.8 (0.7) | 2.3(1.5) | 2.08 | 0.35 | 2.1 (1.3) | 1.8 (1.2) | 1.7(1.1) | 1.63 | 0.44 |
|  |  | False miss errors (Go) | 5.6 (11.5) | 2.2 (3.1) | 3.6(2.6) | 0.90 | 0.64 | 4.1 (8.1) | 3.4 (2.5) | 4.8(12.6) | 1.59 | 0.45 |
|  |  | Reaction time (Go) (ms) | 342.8 (40.0) | 352.8 (41.9) | 386.0(55.1) | 4.22 | 0.12 | 364.8 (56.4) | 369.7 (48.5) | 401.4(68.9) | 2.00 | 0.37 |
| Executive function | Maze | Trials completed | 10.4 (7.7) | 6.7 (3.4) | 7.9(5.2) | 2.69 | 0.26 | 6.8 (2.3) | 6.2 (2.3) | 5.7(2.6) | 1.80 | 0.41 |
|  |  | Total errors | 63.9 (81.6) | 21.4 (12.9) | 17.1(11.9) | **7.60** | **0.02** | 23.3(7.9) | 18.0(11.9) | 11.6(7.2) * | **16.67** | **<0.001** |
| Information processing | Choice Reaction time | Reaction time (s) | 1.3 (1.3) | 0.8 (0.2) | 0.7(0.1) | 4.22 | 0.12 | 0.7 (0.1) | 0.8 (0.3) | 0.8(0.2) | 1.56 | 0.45 |
| Language | Spot the Real Word | Accuracy (%) | 46.0 (6.7) | 44.6 (9.7) | 45.2(10.9) | 1.54 | 0.46 | 43.5 (10.2) | 44.5 (10.3) | 47.3(3.1) | 3.30 | 0.19 |
|  | Word Generation | Number of words generated | 15.9 (6.0) | 17.6 (4.9) | 17.3(4.8) | 2.20 | 0.33 | 13.6 (3.8) | 15.3 (3.2) | 17.4(3.8) | **17.17** | **<0.001** |
|  |  | Animal names generated | 22.3(5.5) | 23.7 (7.9) | 25.5(5.8) | 1.89 | 0.39 | 23.9(5.6) | 26.1 (7.3) | 2575(7.5) | 5.09 | 0.08 |
| Memory | Memory Recall | Total immediate recall | 33.3 (2.5) | 36.2 (2.3) | 38.2(3.4) | **11.82** | **0.003** | 33.7 (6.5) | 37.4 (4.0) | 38.7(5.3) | **8.21** | **0.02** |
|  |  | Long delay recall | 8.2 (2.1) | 9.8 (1.0) | 10.5(1.1) | 5.72 | 0.06 | 7.5 (3.2) | 9.6 (1.8) | 9.9(2.3) | 5.55 | 0.06 |
|  | Memory Recognition | Recognition accuracy | 10.8 (1.2) | 10.9 (1.1) | 11.3(0.8) | 5.16 | 0.08 | 10.8 (1.2) | 10.9 (1.1) | 11.3(0.8) | 5.16 | 0.08 |
|  | Digit Span | Recall span (forwards) | 6.6 (2.0) | 6.6 (0.7) | 7.0(1.4) | 1.81 | 0.41 | 7.3 (1.1) | 6.7 (2.3) | 5.8(2.5) | 1.81 | 0.41 |
|  |  | Recall span (backwards) | 5.5 (1.8) | 5.7 (2.7) | 5.2(2.7) | 2.27 | 0.32 | 5.0 (1.5) | 5.6 (2.1) | 5.6(1.9) | 2.67 | 0.26 |
|  | Span of Visual Memory | Recall span | 5.5 (1.0) | 5.4 (1.4) | 5.6(0.8) | 0.47 | 0.79 | 5.8 (1.3) | 5.7 (0.9) | 5.2(1.5) | 0.51 | 0.77 |
| Sensory-motor | Motor Tapping | Number of taps (dominant) | 179.1 (15.3) | 167.4 (11.7) | 161.0(25.7) | 2.89 | 0.24 | 163.5(31.3) | 169.0 (15.9) | 164.1(24.3) | 0.20 | 0.91 |
|  |  | Number of taps (non-dominant) | 155.0 (16.4) | 153.9 (13.1) | 137.6(27.7) | 1.54 | 0.46 | 148.6 (22.2) | 143.6 (22.9) | 140.3(26.2) | 1.26 | 0.53 |
| Social cognition | Emotion Recognition | Fear accuracy (%) | 83.3 (20.0) | 80.6 (15.5) | 77/8(26.4) | 0.52 | 0.77 | 72.9 (29.1) | 77.1 (20.5) | 82.3(15.5) | 0.17 | 0.92 |
|  |  | Fear reaction time (s) | 3.5 (1.1) | 2.8 (0.9) | 2.3(0.7) | 10.5 | 0.005 | 3.0 (1.0) | 2.6 (0.8) | 2.6(0.5) | 2.67 | 0.26 |
|  |  | Angry accuracy (%) | 65.3 (10.4) | 70.8 (14.0) | 58.3(17.7) | 6.46 | 0.04 | 65.7 (16.3) | 62.5 (18.5) | 59.4(9.4) | 4.62 | 0.10 |
|  |  | Angry reaction time (s) | 2.7 (0.9) | 2.3 (1.1) | 2.1(0.6) | 5.20 | 0.55 | 2.4 (1.1) | 2.0 (0.6) | 2.1(0.5) | 4.17 | 0.13 |
|  |  | Disgust accuracy (%) | 58.3 (15.3) | 52.8 (16.2) | 45.8(18.8) | 1.18 | 0.96 | 52.1 (22.5) | 47.9 (19.8) | 52.1(21.2) | 1.70 | 0.43 |
|  |  | Disgust reaction time (s) | 2.6 (0.9) | 2.3 (0.8) | 2.4(1.0) | 0.51 | 0.77 | 2.4 (0.6) | 2.3 (0.8) | 2.3(0.7) | 0.17 | 0.92 |
|  |  | Sad accuracy (%) | 76.4 (13.2) | 70.8 (31.3) | 54.2(35.4) | 5.07 | 0.08 | 65.6 (15.2) | 70.8 (24.0) | 67.7(19.6) | 2.00 | 0.37 |
|  |  | Sad reaction time (s) | 2.8 (1.3) | 2.2 (0.6) | 2.1(0.5) | 5.540 | 0.06 | 2.8 (1.2) | 2.5 (1.4) | 2.3(1.0) | 3.17 | 0.21 |
|  |  | Happy accuracy (%) | 98.6 (4.2) | 97.2 (5.5) | 94.4(11.0) | 1.27 | 0.53 | 99.0 (3.6) | 100.0 (0) | 94.8(11.3) | 3.50 | 0.17 |
|  |  | Happy reaction time (s) | 1.4(0.2) | 1.4 (0.3) | 1.3(0.2) | 0.22 | 0.90 | 1.4(0.4) | 1.3 (0.2) | 1.3(0.3) | 4.67 | 0.10 |
|  |  | Neutral accuracy (%) | 97.2 (5.5) | 98.6 (4.2) | 85.8(8.8) | 1.07 | 0.58 | 92.7 (9.9) | 93.8 (8.4) | 91.7(11.1) | 0.20 | 0.91 |
|  |  | Neutral reaction time (s) | 1.4 (0.2) | 1.3 (0.2) | 1.2(0.2) | 6.89 | 0.03 | 1.4 (0.7) | 1.8 (1.2) | 1.4(0.3) | 4.17 | 0.13 |

**Exclude one subject with extremely high value of errors at follow-up.* ***Bold*** *indicates significant results after alpha adjustment.*
